# Cost of Pediatric Visceral Leishmaniasis Care in Morocco

**DOI:** 10.1371/journal.pone.0155482

**Published:** 2016-06-03

**Authors:** Nabil Tachfouti, Adil Najdi, Sergi Alonso, Elisa Sicuri, Abderahmane Laamrani El Idrissi, Chakib Nejjari, Albert Picado

**Affiliations:** 1 Laboratory of Epidemiology, clinical Research and Community Health, Faculty of Medicine and pharmacy of Fez, Sidi Mohamed Ben Abdellah University, Fez, Morocco; 2 ISGlobal, Barcelona Ctr. Int. Health Res. (CRESIB), Hospital Clínic—Universitat de Barcelona, Barcelona, Spain; 3 Center of Epidemiology and disease Control, Ministry of Health, Rabat, Morocco; 4 Ecole Nationale de Santé Publique (ENSP), Ministry of Health, Rabat, Morocco; The Australian National University, AUSTRALIA

## Abstract

**Background:**

Visceral leishmaniasis (VL) is a neglected parasitic disease that is fatal if left untreated. VL is endemic in Morocco and other countries in North Africa were it mainly affects children from rural areas. In Morocco, the direct observation of *Leishmania* parasites in bone marrow aspirates and serological tests are used to diagnose VL. Glucantime is the first line of treatment. The objective of this study was to report the costs associated to standard clinical management of pediatric VL from the provider perspective in Morocco. As a secondary objective we described the current clinical practices and the epidemiological characteristics of pediatric VL patients.

**Methods:**

From March to June 2014 we conducted a survey in eight hospitals treating pediatric VL patients in Morocco. A pro-forma was used to collect demographic, clinical and management data from medical records. We specifically collected data on VL diagnosis and treatment. We also estimated the days of hospitalization and the time to start VL treatment. Costs were estimated by multiplying the use of resources in terms of number of days in hospital, tests performed and drugs provided by the official prices. For patients receiving part of their treatment at Primary Health Centers (PHC) we estimated the cost of administering the Glucantime as outpatient. We calculated the median cost per VL patient. We also estimated the cost of managing a VL case when different treatment strategies were applied: inpatient and outpatient.

**Results:**

We obtained data from 127 VL patients. The median total cost per pediatric VL case in Morocco is 520 US$. The cost in hospitals applying an outpatient strategy is significantly lower (307 US$) than hospitals keeping the patients for the whole treatment (636 US$). However the outpatient strategy is not yet recommended as VL treatment for children in the Moroccan guidelines. VL diagnosis and treatment regimens should be standardized following the current guidelines in Morocco.

## Introduction

Visceral leishmaniasis (VL), also known as kala-azar, is a neglected parasitic disease transmitted by sand flies. VL is usually fatal if not treated and causes 20 to 40 thousand deaths per year worldwide [1]. In North African countries VL is caused by *Leishmania infantum* and affects primarily children living in poor rural areas with limited access to diagnostic and treatment.

In Morocco, leishmaniasis is a priority for the Ministry of Health due to their public health and economic impact [2]. VL is endemic in the northern regions but sporadic cases are reported in the South of the country [3]. Over 150 VL cases are reported per year in Morocco [3], however this figure is suspected to be a fraction of the real burden of the disease. Some authors estimated that the VL incidence may be as high as 600 cases per year [1]. In Morocco VL patients are usually below 5 years old and present the triad of splenomegaly, fever and pancytopenia in most of the cases [4–6]. Fever is usually associated with rigor and chills and may be intermittent and irregular. Loss of appetite, pallor, weight loss and weakness are also common in VL patients [4–6].

In Morocco VL is diagnosed based on a combination of clinical signs (e.g. fever >2 weeks) with parasitological and/or serological (Immunofluorescence (IFI) or ELISA) tests in patients coming from endemic areas [2]. The parasitological confirmation involves visualizing *Leishmania* parasites in bone marrow, spleen or lymph node aspirates. The parasitological test, which remains the gold standard, requires an invasive and painful procedure (e.g. sternal aspiration in children) and trained laboratory personnel. The serological tests currently applied also require laboratory facilities and expertise limiting the VL diagnostic capacity to tertiary care hospitals in Morocco. Rapid diagnostic tests (e.g. rk39 dipstick) extensively used in other VL endemic areas [7] are not currently used in Morocco. Meglumine antimoniate (Glucantime) remains the first line treatment for pediatric VL in Morocco. Glucantime, which is not available at private pharmacies, is included in the National Essential Drug List. According to the national guidelines, this treatment (20 mg of pentavalent antimonial (Sb^v^)/kg for 20 days) requires close clinical monitoring [2]. In Morocco the fatality rate of VL patients treated with Glucantime range from 1 to 4% [3,4,6,7]. Liposomal amphotericin B (Ambisome) is the second line of treatment but it is rarely used [2].

In Morocco, despite the relevance of pediatric VL in some areas, the burden of this disease is not well known. Most of the costs associated to VL management are covered by the Ministry of Health (MoH) as medical care is provided for free). As shown in other countries, evaluating the cost of diagnosing and treating leishmaniasis and its financial impact on society may help bringing this disease out of the neglect and trigger research to implement new clinical management options [8,9]. In fact, despite the relatively contained number of cases registered each year, the complicated procedures needed for the detection of VL in endemic areas and the expensive treatment of the severe cases, are likely to imply a relevant economic and financial burden to the Moroccan health system. The objective of this study was to report the costs associated to the clinical management of pediatric VL from the provider perspective in Morocco. As a secondary objective we described the current clinical practices and the epidemiological characteristics of pediatric VL patients. The results of this study in Morocco can be translated to other countries in North Africa facing similar challenges.

## Methods

The costs associated to pediatric VL in Morocco were estimated using patient data gathered from hospital records. Clinical and epidemiological data were also collected from those two sources.

### Hospital records

#### Selection of hospitals

We used the number of reported VL patients per province from 2003 to 2012 to select the 10 provinces with the highest case load in Morocco. The number of VL cases per province was provided by the Ministry of Health. We then selected public hospitals with a pediatric unit located in those provinces or in the surrounding area. We assumed that those hospitals would treat VL patients regularly as they are located in the *L*. *infantum* endemic region in Morocco.

#### Data collection

The selected hospitals were visited from March to June 2014. The available medical records from VL patients treated in each hospital in recent years (2009 to 2014) were retrieved. A pro-forma was used to collect 1) demographic (e.g. age, gender) and 2) clinical and 3) management data. The data collected included information on laboratory (e.g. complete blood count, serum creatinine, serum amylase and electrolytes), diagnostic (e.g. serology and parasitology) and other tests (e.g. x-rays, echography) performed to each patient and drugs used. We collected individual information on the diagnostic procedure (e.g. Bone Marrow Aspiration, ELISA) and treatment (e.g. number of injections of Glucantime) for VL. Relevant dates (e.g. date when symptoms started, date of admission, date when VL treatment started and ended, date of release) were also noted and used to estimate the days of hospitalization and the delay in starting VL treatment after admission.

For each episode of VL, we recorded which tests were performed in the hospital and which were performed in private institutions. When available, the outcome of the VL treatment was also recorded. When the VL treatment was not completed at the hospital, we estimated the number of doses the patient received at the primary health center (PHC) assuming a complete treatment of 20 doses [2].

#### Cost estimation

The information retrieved from records was used to estimate direct costs from the healthcare provider perspective. Individual records included the use of resources for the management of the VL episode. Costs were estimated by multiplying the use of resources in terms of number of days in hospital, tests performed and drugs provided in the public hospital by the official prices (unit costs) published by “L’Agence Nationale de l’Assurance Maladie”[10]. We assumed that the costs of all the drugs prescribed in the hospital were covered by the Ministry of Health. Differences in unitary costs (e.g. cost of hospitalization) between Regional and Provincial (CHR/P) and University (CHU) hospitals were taken into account. Hospitalization cost consisted of equipment, infrastructure, overhead costs, and personnel costs and these were already included in the official rate of day of hospitalization. We calculated the costs of tests conducted in the hospital and those performed in private laboratories or health facilities. To estimate the unit costs of the tests (e.g. VL serology, X-rays) performed in the private sector we used the prices applied in 4 private laboratories in Fez and Taza.

We specifically evaluated the costs of diagnosing and treating VL and differentiate them from other laboratory tests and drugs. VL treatment cost was calculated based on the cost per vial of Glucantime applied at the CHU in Fez: 1.70 US dollars (US$). We assumed that each day of treatment (each injection) required one single use vial of Glucantime. The personnel cost for the administration of Glucantime in the hospital was included in the hospitalization cost. For Glucantime treatments provided at the PHC (outpatient treatment), we assumed a cost of 0.90 US$ per injection, which included the wage of a nurse (5 minutes per treatment). Nurse’s time was valued based on the salary of a state-licensed nurses principal-mid grade (1,733.26 US$/month [11]). Recurrent non-medical and capital costs at the PHC were not taken into account. The complete list of resources and unit costs used in this study is provided as supplementary material (S1 Table).

### Analysis

#### VL patients

Only the data from pediatric patients for whom we had confirmed diagnosis of VL (i.e. bone marrow aspirate and/or serology) and/or a record of anti-leishmanial treatment (i.e. Glucantime injections) were included in the analyses. The demographic (e.g. age, gender) and clinical data were used to describe the study population and the differences in clinical management of VL patients. The clinical data analyzed included the diagnosis and treatment of VL as well as clinical management indicators: days of hospitalization, tests to monitor patients (e.g. ALT, Blood count tests, electrocardiography (ECG), X-rays) and other treatments received (e.g. transfusions, antibiotics). The number of Glucantime doses provided at the PHC were used to determine the treatment strategy used in different hospitals. Hospitals where more than 70% of the patients received most of their VL treatment (more than 60% of the Glucantime doses: 14 to 21 injections) at the PHC were defined as hospitals using an ambulatory or out-patient care strategy. The rest used a hospital based or inpatient strategy. The results were presented per hospital, by treatment strategy (e.g. inpatient vs outpatient) and for the whole study population. Interquartile ranges (IQR) and 95% confidence intervals were calculated and presented when appropriate.

#### VL management costs

All costs were calculated in Moroccan Dirhams (MAD) and later converted to 2014 US$ [12]. Costs were adjusted by applying a discount rate of 3% and corrected by the annual inflation index [13].

The costs of VL management were divided into 5 cost categories: (1) diagnostic VL, (2) treatment VL, (3) Hospitalization, (4) tests performed (e.g. Blood count, ECG) and (5) drugs (other than anti-leishmanial) administered. The costs of VL diagnosis and tests were divided between those conducted in the hospital and those performed in private facilities. The costs of VL treatment were divided between those in the hospital and those in the PHC. Finally, tests and drugs were combined to estimate the mean cost of a day in hospital. This value encompassed all costs of managing a pediatric VL episode in hospital excluding those specifically related to diagnosis and treatment of VL.

All resources used as part of the management of pediatric VL were considered as financial costs covered by the healthcare provider. The diagnostic tests performed at private health facilities, initially paid by households, should be refunded by the national health insurance. Two different cost scenarios were constructed. Scenario 1 considered the use of public resources at their public unit price and the use of private resources at their respective private unit price; and scenario 2 considered both the public and private resources at their public unit price, as if all test done at private health facilities were carried out in public health facilities. Scenario 2 implied that financial and economic costs are equal and represented the cost of VL management if all resources used were conducted in public hospitals. In this manuscript we present the results of scenario 1. Scenario 2 is presented as supplementary material (S3 Table). The cost data is presented as median and inter-quartiles for both VL treatment strategies: inpatient vs outpatient.

#### Statistical analyses

The non-parametric test *Mann-Whitney-Wilcoxon* was used to identify statistically significant differences between inpatient and outpatient strategies. The sample power was retrospectively estimated using bootstrapping methods [14]. Pearson's coefficient of correlation was used to identify association among cost categories.

A univariate sensitivity analysis was carried out to assess the robustness of results by changing values of three key parameters within plausible ranges. We evaluated the changes in the median cost of diagnosing and treating a VL case when (1) the discount rate applied was either 0% or 5%, (2) the cost of a vial of Glucantime varied from 1.2 US$ (official price negotiated by WHO [7]) to 3.4 US$ [15] and (3) when the salary of the nurse administering Glucantime at the PHC ranged from principal-low grade (US$ 1,186.83/month) to principal-high grade (US$ 1,895.00/month) [11]. Modifying the nurse’s salary resulted in varying the cost of administering Glucantime at PHC from US$ 0.62 to US$ 0.99 per treatment. We use a tornado diagram to present the results of the sensitivity analysis. The analyses were performed in stata v10 (StataCorp) and Excel (Microsoft).

### Ethical considerations

The study protocol was approved by the Comité d’éthique du CHU Hassan II in Fes (Morocco) and Comité Ético de Investigación Clínica del Hospital Clínic de Barcelona (Spain). The clinical data from patients were anonymized: no names, addresses or any other information allowing the identification of individuals were recorded.

## Results

### Hospitals surveyed

**Fig 1:**
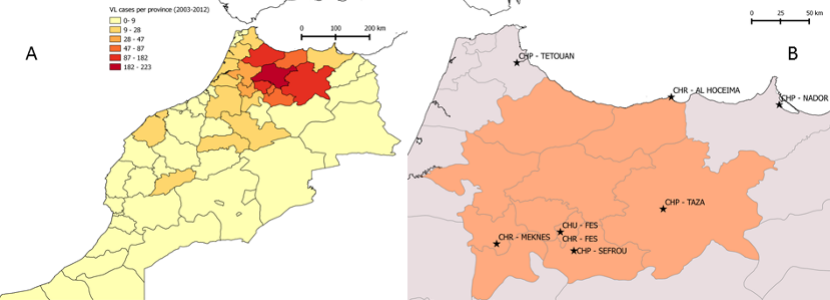
(A) Map of North Morocco showing the distribution of visceral leishmaniasis (VL) cases reported per province from 2003 to 2012. (B) The ten provinces with the highest number of VL cases reported from 2003 to 2012 highlighted in red and the 8 Hospitals selected for the cost study identified with a star. Map generated with QGIS 2.2.0-Valmiera and geographical data from GADM (http://www.gadm.org/).

The ten provinces with the highest VL case load from 2003 to 2012 are presented in Fig 1. Those provinces account for more than 76% (1051/1370) of VL cases reported in Morocco from 2003 to 2012. This is the highest endemic area for VL in Morocco as described by other authors previously [1,3]. Eight hospitals with a pediatric unit were identified in the region (Fig 1). One was a University Hospital (Centre Hospitalier Universitaire–CHU), four were regional (CHR) and three were provincial (CHP) hospitals. We did not find any medical records of VL patients in one of the hospitals, thus the analyses were conducted on data retrieved from the remaining 7.

### VL patients

Out of the 131 clinical records obtained four were excluded as there was no information on diagnosis or treatment of VL. Table 1 details the demographic and VL clinical management of the 127 VL patients finally retained for the analyses. Briefly, the number of clinical records retrieved from the 7 hospitals ranged from 9 to 37. The 127 VL patients had a mean age of 2.9 and half of them were males (49.6%). Based on the data available (n = 97), pediatric patients had mean weight of 11.4 kg (SD = 4.8). The distribution of patients per weight is provided as Supplementary material (S2 Table). Five patients (3.9%) could be considered treatment failures. Of them, four (3.1%) had to receive more than one Glucantime treatment and one (0.8%) died during treatment. However most of the records did not have information on the final outcome. None of the 127 VL cases was treated with Liposomal Amphotericin B, the second line treatment in Morocco.

**Table 1:** Demographic Information and data on diagnosis and treatment procedures to manage pediatric visceral leishmaniasis (VL) in Morocco. Data extracted from 127 clinical records in 7 hospitals in Morocco. Mean and 95% Confidence Intervals (CI), Median and Interquartile Range (IQR) or percentages are used when appropriate.

		Demographic information		Diagnosis Visceral Leishmaniasis			VL Treatment with Glucantime			
		Age (years)	Gender	Serology	Parasitology	Both Tests	Total Doses	Doses Hospital	Doses PHC	Outpatient Patients
Hospital	N	Mean (95% CI)	% males / % females	% (95% CI)	% (95% CI)	% (95% CI)	Median (IQR)	Median (IQR)	Median (IQR)	%
1	12	2.1 (1.3–2.9)	25.0 / 75.0	91.7 (61.5–99.8)	83.3 (51.6–97.9)	75.0 (42.8–94.5)	24.0 (24.0–25.5)	17.0 (12.0–24.0)	9.5 (2.5–13.0)	25%
2	26	3.3 (2.2–4.4)	44.0 / 66.0	73.1 (52.2–88.4)	76.9 (56.4–91.0)	53.8 (33.4–73.4)	21.0 (21.0–21.0)	9.0 (8.0–11.0)	12.5 (11.0–14.0)	27%
3	9	4.5 (1.3–7.8)	55.6 / 44.4	66.7 (29.9–92.5)	44.4 (13.7–78.8)	33.3 (7.5–70.1)	21.0 (21.0.21.0)	20.0 (10.0–21.0)	0.0 (0.0–11.0)	11%
4	13	2.8 (2.0–3.6)	33.3 / 66.7	30.8 (9.1–61.4)	92.3 (64.0–99.8)	23.1 (5.0–53.8)	20.0 (20.0–21.0)	20.0 (20.0–21.0)	0.0 (N/A)	0%
5	13	3.5 (1.4–5.6)	61.5 / 38.5	92.3 (64.0–99.8)	7.7 (0.2–36.0)	0.0 (N/A)	34.0 (28.0–34.0)	34.0 (28.0–34.0)	0.0 (0.0–0.0)	15%
6	37	2.3 (1.6–2.9)	51.4 / 48.6	29.7 (15.9–47.0)	86.5 (71.2–95.5)	21.6 (9.8–38.2)	21.0 (21.0–21.0)	5.0 (3.0–7.0)	16.0 (14.0–18.0)	86%
7	17	2.8 (1.3–4.2)	73.3 / 26.7	17.6 (3.8–43.4)	70.6 (44.0–89.7)	11.8 (1.5–36.4)	21.0 (21.0–21.0)	6.0 (2.0–9.0)	15.0 (12.0–19.0)	71%
**Total**	127	2.9 (2.4–3.3)	49.6 / 50.4	52.0 (42.9–60.9)	71.7 (63.0–79.3)	30.7 (22.8–39.5)	21.0 (21.0–21.0)	9.0 (6.0–20.0)	12.0 (0.0–17.0)	45%
**Strategy**										
Inpatient	73	3.2 (2.5–3.8)	43.7 / 56.3	71.2 (59.4–81.2)	64.4 (52.3–75.3)	39.7 (28.5–51.9)	21.0 (21.0–24.0)	16.0 (9.0–21.0)	8.0 (0.0–13.0)	18%
Outpatient	54	2.4 (1.8–3.0)	57.7 / 42.3	25.9 (15.0–39.7)	81.5 (68.6–90.7)	18.5 (9.3–31.4)	21.0 (21.0–21.0)	5.0 (3.0–7.0)	16.0 (14.0–18.0)	81%
*MWW* P-value In vs out-patient		0.0166	0.1257	<0.0001	0.0353	0.0107	0.0080	<0.0001	<0.0001	
Test power		67.2%	32.4%	99.9%	55.4%	74.3%	77.9%	100%	100%	

Note: Mann-Whitney-Wilcoxon (MWW) null hypothesis of no differences between inpatient and outpatient strategies.

The diagnosis and treatment of VL also varied in the 7 hospitals included in the study. The bone marrow aspirate was used as diagnostic tool in 71.7% of the patients but in some hospitals this diagnostic method was only used in less than 10% of the patients (e.g. 7.7% in hospital number 5). Serological tests were used in half of the patients (52.0%), but its implementation varied with the hospital (range 17.6 to 92.3%). Finally both serological and parasitological tests were used to diagnose VL in almost one-third (30.7%) of the patients. None of the 7 hospitals had the capacity to conduct serological tests for leishmaniasis at the time of the survey. All serological tests were conducted in private laboratories and were paid by the patient’s relatives. Out of the 66 patients with serological tests, 59 had a result in the clinical records and 58 of them were positive (98%). The serological tests used were ELISA (65%) or IFI (33%). Rapid diagnostic tests were not applied in any of patients. The majority of parasitological tests (64% - 58/91) were conducted in the hospital. The rest (31/91–34%) of the patients had their bone marrow aspirates tested in private laboratories and 2 patients had parasitological results from both the hospital and private laboratories. The majority of the records (80/91) had information on the results of the parasitological test; 87.5% of them (70/80) were positive. In 33 patients we had both serological and parasitological results.

The total number of Glucantime doses was calculated assuming that patients treated in the PHC received the complete treatment. Thus most of the patients in the study received 20 to 21 doses in total. This may be a conservative estimate as there were two hospitals where patients received more doses of Glucantime (e.g. 24 and 34 in hospitals 1 and 5 respectively). In these hospitals Glucantime treatment was longer as the drug was given at progressive doses for 3 to 6 days before starting the standard treatment at 20 mg Sb^v^/kg. In one of these hospitals, Glucantime at therapeutic doses was usually given for 28 days instead of 20 as indicated in the guidelines. When the 127 records were considered, patients seem to receive more Glucantime doses at the PHC (median = 12) than in the hospital (median = 9). However these figures were highly dependent on the strategy used by the hospital. In hospitals using an inpatient approach (hospitals 1 to 5), most of the Glucantime doses were provided in the hospital (median = 16). In hospitals applying an outpatient strategy, only one fifth (median = 5) of the doses were given at the hospital.

The strategy applied to treat VL patients had an impact on the clinical management indicators presented in Table 2. The total days of hospitalization, days from admission to treatment and days of treatment were significantly higher in hospitals using an inpatient approach compared to those applying an outpatient strategy. Similarly, the number of tests (e.g. blood counts, liver and kidney function tests), number of blood transfusions per patient and the proportion of patients receiving antibiotics were significantly higher in hospitals using an inpatient approach. These differences were statistically significant (Table 2). The majority of patients (85/127–67%) had to conduct at least one test in a private laboratory. This proportion increases to 77% (98/127) if diagnostic tests for VL are also considered. These factors have an impact on the monitoring of VL patients under treatment and the costs associated to the clinical management of VL.

**Table 2:** Demographic and clinical information extracted from the clinical records of 127 visceral leishmaniasis (VL) patients in Morocco. Data related to the clinical management of VL patients: days in hospital, days of VL treatment, number (Num.) of tests (e.g. blood counts, ALT or BUN) and blood transfusions per patients as well as the proportion of patients receiving antibiotics are presented. These are just a fraction of the parameters considered in the cost analysis. The data is presented aggregated (1) per hospital, (2) for the whole population and (3) by treatment strategy (inpatient vs outpatient). Mean and 95% Confidence Intervals (CI) or Median and Interquartile Range (IQR) are used when appropriate. Hospitals are identified with numbers.

		Days in hospital	Days to treatment	Days of treatment	Num. Blood Counts	Num. ALT^1^ tests	Num. BUN^2^ tests	Num. Transfusions	Patients with antibiotics
Hospital	N	Median (IQR)	Median (IQR)	Median (IQR)	Mean (95% CI)	Mean (95% CI)	Mean (95% CI)	Mean (95% CI)	% (95% CI)
1	12	28.5 (19.5–31.0)	9.5 (6.0–13.0)	16.5 (12.0–24.5)	11.4 (6.6–16.3)	6.0 (2.3–9.7)	9.1 (2.0–16.2)	0.9 (0.6–1.0)	100.0 (73.5–100.0)
2	26	15.0 (12.0–19.0)	no data	no data	3.1 (2.6–3.6)	1.4 (1.1–1.7)	1.5 (1.3–1.8)	0.8 (0.6–0.9)	88.5 (69.8–97.6)
3	9	20.0 (15.0–25.0)	3.0 (0.0–5.0)	20.0 (9.0–22.0)	1.1 (0.3–1.9)	0.4 (0.0–0.8)	0.1 (0.0–0.4)	0.4 (0.1–0.8)	44.4 (13.7–78.8)
4	13	23.0 (22.0–25.0)	3.0 (2.0–7.0)	19.0 (17.5–20.0)	1.9 (1.2–2.6)	0.3 (0.0–0.7)	0.5 (0.1–0.9)	0.7 (0.4–0.9)	76.9 (46.2–95.0)
5	13	34.0 (31.0–40.0)	5.0 (4.0–7.0)	32.0 (27.0–33.0)	1.8 (1.2–2.4)	2.0 (1.3–2.7)	1.8 (1.1–2.6)	0.2 (0.1–0.5)	61.5 (31.6–86.1)
6	37	6.0 (3.0–8.0)	1.0 (1.0–2.0)	4.0 (2.0–6.0)	0.9 (0.7–1.1)	0.1 (0.0–0.1)	0.2 (0.1–0.4)	0.4 (0.2–0.6)	24.3 (11.8–41.2)
7	17	7.0 (2.0–21.0)	1.0 (1.0–2.0)	7.0 (2.0–20.0)	1.7 (1.1–2.3)	0.2 (0.0–0.6)	0.5 (0.1–0.9)	0.4 (0.1–0.6)	64.7 (38.3–85.8)
**Total**	127	14.0 (7.0–23.0)	2.0 (1.0–6.0)	8.0 (4.0–20.0)	2.7 (2.0–3.3)	1.2 (0.7–1.6)	1.6 (0.8–2.3)	0.5 (0.5–0.6)	60.6 (51.6–69.2)
**Strategy**									
Inpatient	73	21.0 (15.0–30.0)	5.5 (2.0–8.0)	20.0 (12.0–31.0)	3.8 (2.7–4.9)	1.9 (1.2–2.7)	2.5 (1.2–3.7)	0.7 (0.5–0.8)	78.1 (66.9–86.9)
Outpatient	54	6.0 (3.0–9.0)	1.0 (0.0–2.0)	4.0 (2.0–7.0)	1.1 (0.9–1.4)	0.1 (0.0–0.2)	0.3 (0.1–0.5)	0.4 (0.3–0.5)	37.0 (24.3–51.3)
*MWW*P-value In vs out-patient		<0.0001	<0.0001	<0.0001	<0.0001	<0.0001	<0.0001	<0.0001	<0.0001
Test Power		100%	100%	100%	100%	100%	100%	93.7%	99.9%

Note: Mann-Whitney-Wilcoxon (MWW) null hypothesis of no differences between inpatient and outpatient strategies. 1 alanine aminotransferase (ALT) test to evaluate liver function. 2 blood urea nitrogen (BUN) test to evaluate kidney function.

### VL management costs

As shown in Table 3, the median cost of managing a pediatric VL case in Morocco was US$ 520 (mean of US$ 594). More than half of it (US$ 262) corresponded to hospitalization costs which were strongly correlated to the number of days in hospital (Pearson correlation coefficient of 0.98). Fifteen percent of the costs were related to diagnosis and treatment of VL (US$ 87). The rest (33%) were costs related to treatments and tests not directly attributable to VL (US$ 133). The majority of the costs (90%) derived from the use of public resources, most of them in the hospital (US$ 440) and only a 5% at the PHC (US$ 31). The other 10% of the costs corresponded to out-of-pocket expenditures initially paid by the patients’ relatives (US$ 49). These costs are in theory reimbursed by the national health insurance.

**Table 3:** Costs of pediatric visceral leishmaniasis (VL) care in Morocco (US$). Total costs and costs divided by category (VL diagnosis, VL treatment, Hospitalization, Tests and other Treatments) presented for the whole study population and per treatment strategy: inpatient and outpatient. Mean, standard deviation (sd), median, inter-quartile range (IQR) are presented for each category, as well as p-values associated to Mann-Whitney-Wilcoxon (MWW) test and bootstrapping results for assessing the level of power on the comparisons.

	Total (N = 127)					Inpatient (N = 73)					Outpatient (N = 54)						
	Mean	(sd)	Median	(IQR)	%	Mean	(sd)	Median	(IQR)	%	Mean	(sd)	Median	(IQR)	%	MWW Pvalue	Test power
**VL diagnosis**	**42**	**(26)**	**38**	**(22–58)**	**7%**	**51**	**(27)**	**40**	**(36–75)**	**6%**	**30**	**(20)**	**23**	**(21–40)**	**10%**	<0.001	100%
Hospital	11	(12)	0	(0–22)	2%	5	(9)	0	(0–0)	0%	19	(11)	22	(21–23)	6%		
Private	31	(30)	36	(0–40)	5%	46	(29)	39	(36–75)	6%	11	(17)	0	(0–36)	4%		
**VL treatment**	**49**	**(10)**	**49**	**(45–53)**	**8%**	**47**	**(12)**	**47**	**(39–52)**	**6%**	**51**	**(5)**	**52**	**(49–54)**	**16%**	0.003	84.2%
Hospital	21	(16)	15	(10–34)	3%	29	(16)	27	(15–36)	4%	11	(9)	9	(5–13)	4%		
PHC	27	(19)	31	(0–44)	5%	18	(18)	21	(0–34)	2%	40	(13)	43	(37–49)	12%		
**Hospitalization**	**309**	**(236)**	**262**	**(161–376)**	**52%**	**415**	**(251)**	**357**	**(273–464)**	**52%**	**165**	**(99)**	**154**	**(95–214)**	**52%**	<0.001	100%
**Tests**	**148**	**(369)**	**75**	**(30–128)**	**25%**	**228**	**(472)**	**116**	**(84–186)**	**29%**	**40**	**(34)**	**35**	**(13–60)**	**12%**	<0.001	100%
Hospital	120	(351)	48	(12–97)	20%	187	(452)	85	(39–142)	24%	28	(26)	23	(7–45)	9%		
Private	28	(39)	13	(0–40)	5%	40	(45)	22	(4–64)	5%	11	(18)	0	(0–17)	3%		
**Other treatment**	**47**	**(40)**	**58**	**(4–73)**	**8%**	**57**	**(41)**	**64**	**(8–87)**	**7%**	**34**	**(34)**	**21**	**(3–62)**	**10%**	<0.001	96.9%
**Total Costs**	**594**	**(544)**	**520**	**(316–658)**		**798**	**(637)**	**636**	**(548–825)**		**319**	**(134)**	**307**	**(216–416)**		<0.001	100%
Cost of a day in hospital*	**33**	**(13)**	**32**	**(22–40)**		**29**	**(10)**	**28**	**(19–38)**		**39**	**(15)**	**36**	**(29–45)**		<0.001	97.3%

Note: VL treatment at the PHC consisted of the price of Glucantime and the personnel cost of the injection; capital and recurrent costs could not be estimated. VL treatment at the hospital included only the price of Glucantime as personnel, capital and recurrent costs were included in the category “hospitalization”.

*This is the cost of a day in hospital excluding the costs of the diagnostic tests (e.g. serology, bone marrow aspirate) and treatments (e.g. glucantime) specific to visceral leishmaniasis management.

Cost differed with the VL treatment strategy. The total cost, as well as costs of VL diagnosis, hospitalization and other treatments were significantly lower in hospitals applying an outpatient strategy compared to inpatient hospitals (Table 3). The median cost per patient in hospitals following an inpatient approach increased a 22% (US$ 636) compared to the pooled estimate. The cost per patient using an outpatient strategy was 41% lower (US$ 307). VL diagnosis costs were higher in inpatient hospitals (US$ 40) as the large majority of tests to diagnose VL were conducted in private facilities. In contrast in hospitals applying an outpatient approach the VL diagnosis was mainly done in hospital facilities and the overall cost of this category was lower (US$ 23). The treatment of VL was higher in hospitals were patients received most of the treatment at the PHC (US$ 52) compared to inpatient hospitals (US$ 47) as the cost of VL treatment at PHC included the cost of administering Glucantime. The hospitalization costs were more than two times higher in inpatient (US$ 357) than outpatient (US$ 154) hospitals. However, in both strategies these costs represent 52% of the total costs. The cost of tests (other than VL diagnosis) represented 24% in inpatient care compared to 12% using outpatient approach. Bootstrap methods confirmed that results assessed the highest level of power on all comparisons undertook (Table 3).

The median cost of a day in hospital was US$ 32 (mean of US$ 33) when the data from all hospitals was pooled together. This cost was significantly higher in hospitals following an outpatient approach US$ 36 (mean of US$ 39) compare to inpatient hospitals US$ 28 (mean of US$ 29) (Table 3).

When the use of all private resources was estimated at public unit price (scenario 2) the total median cost per VL patient was US$ 486 (mean of US$ 571). The differences between VL treatment strategies were similar to those observed in scenario 1 (S3 Table).

**Fig 2:**
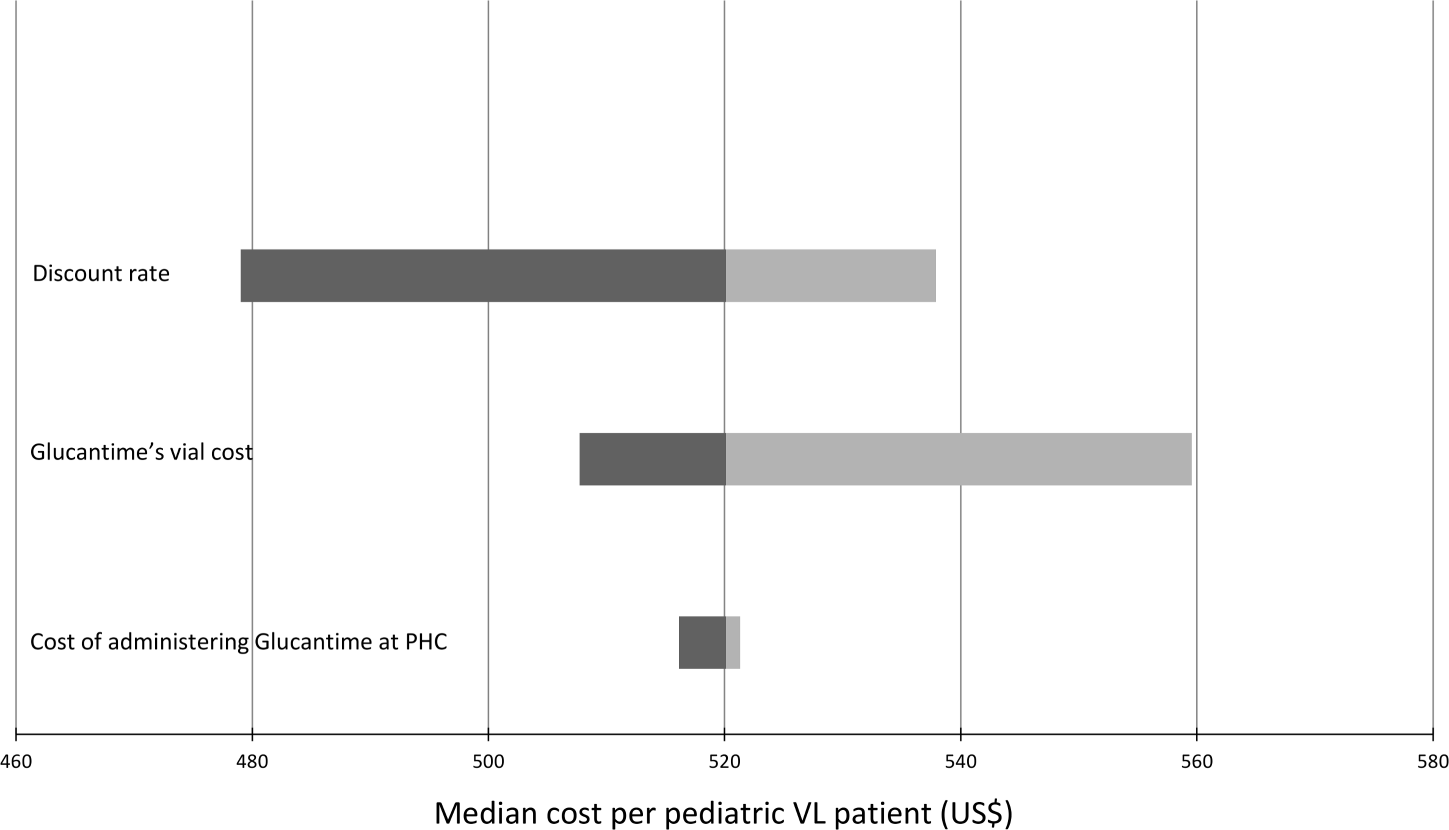
Tornado diagram presenting the results of the univariate sensitivity analysis. Changes on the median cost per pediatric Visceral Leishmaniasis (VL) patient were evaluated by shifting (1) the discount rate (from 0% to 5%, base case %), (2) the cost of a vial of Glucantime (from US$ 1.2 to US$ 3.4, base case US$ 1.70) and (3) the cost of administering Glucantime at the Primary Health Centre (PHC) (from US$ 0.62 to US$ 0.99, base case US$ 0.90).

The sensitivity analysis (Fig 2) showed that modifying the salary of the nurse at the PHC had a limited impact on the median cost per VL episode. However, doubling the cost of Glucantime increased the cost per VL case to US$ 560. Reducing the discount rate from 3% to 0% would increase the median cost per VL episode to US$ 538, while a discount rate of 5% would reduce the costs to US$ 479.

## Discussion

The median cost of diagnosing and treating a pediatric case of visceral leishmaniasis in Morocco is US$ 520. Our estimate is lower than the cost of treating a pediatric VL patient with Glucantime in Greece (935.6 to 1639 euros) [16] or in Italy (over 3000 euros) [17]. To our knowledge there are no other cost studies for VL in North Africa. In Morocco, the studies on the cost of infectious diseases are scarce. Using a chronic disease as a reference; the cost of lung cancer management in Morocco is estimated to range between US$ 3,420 and 4,600 per year [18]. The limited cost of VL management and the low prevalence of this disease in Morocco should allow providing the best standards of care to all children suffering from VL.

An increase in discount rate from 3% to 5% and an increase in Glucantime price for injection from 1.2 to 3.4 US$) had an impact higher than 5% over the total median cost per VL case. All other variations applied to parameters for sensitivity analysis led to shifts in the median costs lower than 5%. For example, adopting no discount (discount rate of 0%) implied a cost increase of about 3.5%. The small variation could be explained by the relatively short time horizon of the analysis, 6 years.

The economic burden of VL management from a societal perspective is likely to be significantly greater as the households costs associated to VL treatment (e.g. indirect medical costs, transport, loss of income to the attendant), not included in the current analysis, may be relevant [9]. In Morocco the diagnosis and treatment are covered by the MoH but this study shows that in some hospitals some tests (e.g. blood count, x-rays, ECG) are conducted outside the hospital facilities. In particular, none of the hospitals in the study had the capacity to conduct serological tests for VL. This increased the cost of VL management as patient’s had to submit their samples or conduct their tests in external private laboratories. If those tests were carried out in public health facilities (scenario 2) the median cost per VL patient would be US$ 486.

More importantly our study shows that two VL treatment strategies are applied in Morocco. Most of the patients receive their complete VL treatment in the hospital but a significant number of VL cases receive part of their Glucantime injections as outpatients in the PHC. These different approaches have a profound effect on the cost of VL management as the outpatient strategy reduces significantly, among others, the hospitalization costs. It also reduces de costs associated to laboratory and other tests (e.g. blood count, hepatic function) which are related to the monitoring of the VL cases. The outpatient strategy, which is less expensive to the provider (US$ 307) has been suggested as an alternative to inpatient VL treatments in children in Greece [9]. A small study (n = 20 children) showed that the outpatient care seems to be safe and effective [19] and 27% cheaper (1639 vs 935.6 euros) than the inpatient strategy [16]. However pediatricians in Italy are against the outpatient administration of antimonials as it requires a close monitoring (e.g. fatal toxicities) [17]. Furthermore, this strategy is not recommended as VL treatment for children in the WHO [7] or the Moroccan [2] guidelines. The efficacy and safety of outpatient strategy in Morocco should be evaluated. Until then, the inpatient strategy recommended by the national guidelines [2], which is 35% more expensive (US$ 636), may be used as a reference.

Our study reports other important differences between hospitals: total days of hospitalization, time to VL diagnosis, duration of the treatment, etc. Some of these differences are due to variability among cases (e.g. severe cases require longer hospitalizations, closer monitoring) but others are related to differences in management and hospital facilities. For example, none of the hospitals could diagnose VL with serological tests but hospitals that have an easy access to MoH laboratories diagnosing VL from BM aspirates (e.g. Hospital number 6) have a reduced time to VL diagnosis (median 1 day). In some hospitals pediatricians apply treatment regimens not recommended by the national guidelines (e.g. progressive doses, 28 days treatment) which increase the days in hospital. It should be noticed that the recommended treatment for VL in Morocco is a shorter (Glucantime for 20 days) than the regimen suggested by the WHO (Glucantime for 28 days) [7]. This shorter regimen was incorporated in the national guidelines in 2010 after an expert consultation led by the Ministry of Health (Laamrani personal communication). Longer treatments of Glucantime (up to 28 days) can be administered if necessary [2]. In this study we did not compare the cost of short (20 doses) and longer (28 doses) glucantime treatments. Data on efficacy (e.g. relapses at 6 months) which are currently not available in most of the cases would allow estimating the cost-effectiveness of the different treatments and strategies. Access to VL diagnosis and treatment regimens should be standardized following the current guidelines in Morocco [2].

This cost study is based on a cohort of 127 VL patients treated in 7 hospitals. These hospitals were selected as they are in the VL endemic area in Morocco [3]. However there are sporadic VL cases reported in other provinces e.g. 24% (n = 319) of the VL cases recorded from 2003 to 2012. Those patients may be diagnosed and treated in hospitals not included in our study. As we could not visit all the hospitals in Morocco we selected those serving the VL endemic area. It is difficult to predict the effect of including hospitals from other provinces in our cost estimates. Those hospitals may diagnose and treat VL cases sporadically. The number of cases (n = 127) included in the study approximately corresponds to the annual incidence of VL in Morocco. The mortality and treatment failure estimates from this cohort (0.8% and 3.1% respectively) are similar to those reported in case series studies in Morocco and other countries in the area [5,6]. However they need to be interpreted with caution as information on the outcome was missing in all the cases referred to the PHC for outpatient treatment and none of the cases records evaluated had follow-up information at 6 months post-treatment as required by WHO guidelines [7]. The number of medical records retrieve per hospital was also variable. Some had an efficient records system (e.g. Hospital number 2) but others were less well organized, e.g. one hospital had to be excluded as no VL records were found. Improving and standardizing the hospital records archives and databases would allow conducting retrospective studies more efficiently. Despite some information was missing in the registries, the main strength of this cost study is the use of patient level data which allowed micro-costing, a more precise approach compared with others (top-down for instance) through which variability of resources used is well represented. As a result, our study provides valuable economic estimates as well as clinical and management information. These data should help health managers in Morocco and other countries in the region improving the management of VL in children.

## Supporting Information

S1 Table Unit costs associated to resources used by pediatric visceral leishmaniasis (VL) patients in Morocco (US$). (DOCX)

S2 Table Distribution of pediatric visceral leishmaniasis (VL) per weight category in Morocco (n = 97). (DOCX)

S3 Table Costs of pediatric visceral leishmaniasis (VL) care in Morocco (US$). Costs per category as private resources were charged at a public unit price (SCENARIO 2). Total costs and costs divided by category (VL diagnosis, VL treatment, Hospitalization, Tests and other Treatments) presented for the whole study population and per treatment strategy: inpatient and outpatient. Mean, standard deviation (sd), median, inter-quartile range (IQR) are presented for each category.
(DOCX)
