# Supplementary material for: Cost of Pediatric Visceral Leishmaniasis Care in Morocco
Source: PLoS One. 2016 Jun 3;11(6):e0155482. doi: 10.1371/journal.pone.0155482 (PMC4892465; doi:10.1371/journal.pone.0155482)
Supplement: S1 Table — (DOCX) [file pone.0155482.s001.docx]

**Supplementary material**

S1 Table: Unit costs associated to resources used by pediatric visceral leishmaniasis (VL) patients in Morocco (US$).

| **HOSPITALIZATION COST** | | | | | |
| --- | --- | --- | --- | --- | --- |
| **Concept** | **CHR/CHP** | | **CHU** | **Comments** | |
| 1 to 3 nights | 29.73 | | 35.67 | Cost per night, the first 3 nights | |
| 4 to 10 nights | 17.84 | | 35.67 | Cost per night, from 4^th^ to 10^th^ night | |
| More than 10 nights | 11.89 | | 35.67 | Cost per night, from 10^th^ night onwards | |
| **VL DIAGNOSTIC COST** | | | | | |
| **Concept** | **CHR/CHP** | | **CHU** | **Private** | **Comments** |
| Serology | 11.70 | | 11.70 | 35.67 |  |
| Bone Marrow | 21.40 | | 21.40 | 39.60 |  |
| **VL TREATMENT COST** | | | | | |
| **Concept** | **CHR/CHP/CHU** | | **PHC** | **Comments** | |
| Glucantime injection (best estimate) | 1.70 | | 1.70 | Source: Ministry of Health | |
| Glucantime injection (minimum) | 1.20 | | 1.20 | Source: *La lute contre les leishmanioses*, WHO 2010. | |
| Glucantime injection (maximum) | 3.40 | | 3.40 | 2013 market price (http://erc.msh.org/) | |
| Median state nurse hourly wage | - | | 10.83 | Source: Ministère de la fonction publique | |
| Monthly salary of a state-licensed nurse principal-mid grade | - | | 1,733.26 | Source: Ministère de la fonction publique | |
| Monthly salary of a state-licensed nurse principal-low grade | - | | 1,186.83 | Source: Ministère de la fonction publique | |
| Monthly salary of a state-licensed nurse principal-high grade | - | | 1,895.00 | Source: Ministère de la fonction publique | |
| **OTHER DIAGNOSTIC TESTS COST** | | | | | |
| **Concept** | **CHR/CHP** | **CHU** | | **Private** | **Comment** |
| Transfusion | 57.07 | 42.81 | | - |  |
| NFS | 8.56 | 8.56 | | 9.51 |  |
| Vitesse Sédimentation | 3.21 | 3.21 | | 3.57 |  |
| Electroph Proteines Sérique | 10.70 | 10.70 | | 29.73 |  |
| Glucose | 3.21 | 3.21 | | 3.57 |  |
| Uree | 3.21 | 3.21 | | 3.57 |  |
| Créatinine | 3.21 | 3.21 | | 3.57 |  |
| Acide Urique | 3.21 | 3.21 | | 3.57 |  |
| Triglyceride | 6.42 | 6.42 | | 5.95 |  |
| Cholesterol total | 3.21 | 3.21 | | 3.57 |  |
| Cholesterol HDL | 3.21 | 5.35 | | 5.95 |  |
| Cholesterol LDL | 3.21 | 3.21 | | 5.95 |  |
| Bilirubine total | 7.49 | 7.49 | | 10.11 |  |
| Bilirubine liée | 7.49 | 7.49 | | 10.11 |  |
| Bilirubine conjuguée | 7.49 | 7.49 | | 10.11 |  |
| Bilirubine non-conjuguée | 7.49 | 7.49 | | 10.11 |  |
| Bilirubine neonatal | 7.49 | 7.49 | | 10.11 |  |
| Bilirubine directe | 7.49 | 7.49 | | 10.11 |  |
| Transaminases P (ALAT GPT) | 5.35 | 5.35 | | 5.95 |  |
| Transaminases O (ALAT GOT) | 5.35 | 5.35 | | 5.95 |  |
| Gamma glutamyltransférase (GGT) | 5.35 | 5.35 | | 7.13 |  |
| Phosphatases alcalines (PAL) | 5.35 | 5.35 | | 7.13 |  |
| Calcium | 3.21 | 3.21 | | 4.76 |  |
| Phosphore | 3.21 | 4.28 | | 4.76 |  |
| Fer serique | 6.42 | 6.42 | | 8.32 |  |
| Sodium | 3.21 | 3.21 | | 3.57 |  |
| Potassium | 3.21 | 3.21 | | 3.57 |  |
| Chlore | 3.21 | 3.21 | | 4.16 |  |
| Resérve alkaline | 3.21 | 3.21 | | 5.35 |  |
| Protide (protein totales) | 3.21 | 3.21 | | 4.76 |  |
| Taux de prothrombine | 4.28 | 4.28 | | 3.57 |  |
| CRP | 10.70 | 10.70 | | 11.89 |  |
| Ecographie abdominal | 23.78 | 42.81 | | 47.56 |  |
| Radiographie | 14.27 | 14.27 | | 23.78 |  |
| ECG | 5.95 | 5.95 | | 11.89 |  |
| Dosage Vit B12 | 35.67 | 42.81 | | 47.56 |  |
| Cytologie culture identification | 9.63 | 9.63 | | 10.70 |  |
| Ecographie cardio | 35.67 | 59.45 | | 77.29 |  |
| Ferritine | 26.75 | 26.75 | | 11.89 |  |
| Fibrinogène | 4.28 | 4.28 | | 4.76 |  |
| Groupe ABD et rhésus | 6.42 | 6.42 | | 7.13 |  |
| LCR | 26.75 | 26.75 | | 29.73 |  |
| Réticulocytes | 3.21 | 3.21 | | 3.57 |  |
| Hépatite A | 26.75 | 26.75 | | 29.73 |  |
| Hépatite B | 64.21 | 64.21 | | 71.34 |  |
| Hépatite C | 32.10 | 32.10 | | 35.67 |  |
| Herpes virus | 21.40 | 21.40 | | 23.78 |  |
| HIV | 21.40 | 21.40 | | 23.78 |  |
| Rubéole | 16.05 | 16.05 | | 17.84 |  |
| Syphilis | 12.84 | 12.84 | | 14.27 |  |
| Toxoplasmose | 16.05 | 16.05 | | 17.84 |  |
| Salmonellose | 8.56 | 8.56 | | 9.51 |  |
| Sideremie | 5.35 | 6.42 | | 7.13 |  |
| Temps de céphaline kaolin | 4.28 | 4.28 | | 4.76 |  |
| Temps de coagulation activée | 5.35 | 5.35 | | 5.95 |  |
| **OTHER TREATMENTS COST** | | | | | |
| **Concept** | **Public price** | | **Comment** | | |
| Abboticine | 3.19 | |  | | |
| Amoxil | 1.86 | |  | | |
| Ampicillline | 4.37 | |  | | |
| Aspegic | 6.78 | |  | | |
| Astaph | 2.37 | |  | | |
| Augmentin | 9.50 | |  | | |
| Aximycine | 1.19 | |  | | |
| Bactrim | 3.35 | |  | | |
| Biosel | 1.45 | |  | | |
| Biotic plus | 14.46 | |  | | |
| Brufen | 2.68 | |  | | |
| Butamyl | 2.54 | |  | | |
| Cacitvitamine D3 | 13.09 | |  | | |
| Calmixene | 5.70 | |  | | |
| Caftriaxonemylan | 32.10 | |  | | |
| Ciproxine | 31.39 | |  | | |
| Clartec | 3.15 | |  | | |
| Cloprame | 1.09 | |  | | |
| Paracetamol | 2.10 | |  | | |
| Cortancyl | 1.59 | |  | | |
| Diapharm | 4.73 | |  | | |
| Doliprane | 1.47 | |  | | |
| Efferalgan | 2.24 | |  | | |
| Fasigyne | 7.88 | |  | | |
| Flagyl | 3.91 | |  | | |
| Fortum | 8.74 | |  | | |
| Fumafer | 3.09 | |  | | |
| Genta | 11.52 | |  | | |
| Glucose 10% | 2.62 | |  | | |
| Glucose 5% | 4.03 | |  | | |
| Konakion | 5.32 | |  | | |
| Lasilix | 0.88 | |  | | |
| Maltofer | 4.24 | |  | | |
| Neomox | 5.46 | |  | | |
| Novoclin | 9.94 | |  | | |
| Perfalgan | 33.06 | |  | | |
| Peridys | 4.41 | |  | | |
| Potassium | 2.93 | |  | | |
| Primperan | 3.35 | |  | | |
| Rhesonativ | 68.61 | |  | | |
| Smecta | 6.23 | |  | | |
| Streptocid | 4.64 | |  | | |
| Tobradex | 4.24 | |  | | |
| Triaxon | 19.73 | |  | | |
| Ultra-levure | 4.53 | |  | | |
| Valium | 3.41 | |  | | |
| Voltarene | 1.39 | |  | | |
