# Supplementary material for: Cost of Pediatric Visceral Leishmaniasis Care in Morocco
Source: PLoS One. 2016 Jun 3;11(6):e0155482. doi: 10.1371/journal.pone.0155482 (PMC4892465; doi:10.1371/journal.pone.0155482)
Supplement: S2 Table — (DOCX) [file pone.0155482.s002.docx]

S2 Table: Distribution of pediatric visceral leishmaniasis (VL) per weight category in Morocco (n=97).

| Weight (kg) | N | Frequency |
| --- | --- | --- |
| < 7.5 | 7 | 7% |
| 7.6-10 | 46 | 47% |
| 10.1-12.5 | 23 | 24% |
| 12.6-15 | 11 | 11% |
| 15.1-17.5 | 3 | 3% |
| 17.6-20 | 4 | 4% |
| > 22.5 | 3 | 3% |
|  | | |
