# Supplementary material for: Cost of Pediatric Visceral Leishmaniasis Care in Morocco
Source: PLoS One. 2016 Jun 3;11(6):e0155482. doi: 10.1371/journal.pone.0155482 (PMC4892465; doi:10.1371/journal.pone.0155482)
Supplement: S3 Table — Costs per category as private resources were charged at a public unit price (SCENARIO 2). Total costs and costs divided by category (VL diagnosis, VL treatment, Hospitalization, Tests and other Treatments) presented for the whole study population and per treatment strategy: inpatient and outpatient. Mean, standard deviation (sd), median, inter-quartile range (IQR) are presented for each category. (DOCX) [file pone.0155482.s003.docx]

S3 Table: Costs of pediatric visceral leishmaniasis (VL) care in Morocco (US$). Costs per category as private resources were charged at a public unit price (SCENARIO 2). Total costs and costs divided by category (VL diagnosis, VL treatment, Hospitalization, Tests and other Treatments) presented for the whole study population and per treatment strategy: inpatient and outpatient. Mean, standard deviation (sd), median, inter-quartile range (IQR) are presented for each category.

|  | Total (N=127) | | | |  |  | Inpatient (N=73) | | | |  |  | Outpatient (N=54) | | | |  | |
| --- | --- | --- | --- | --- | --- | --- | --- | --- | --- | --- | --- | --- | --- | --- | --- | --- | --- | --- |
|  | Mean | (s.d.) | Median | (IQR) | % |  | Mean | (s.d.) | Median | (IQR) | % |  | Mean | (s.d.) | Median | (IQR) | | % |
| **VL diagnosis** | **23** | **(13)** | **22** | **(13-33)** | **4%** |  | **24** | **(13)** | **22** | **(13-33)** | **3%** |  | **23** | **(12)** | **22** | **(21-24)** | | **7%** |
| **VL treatment** | **49** | **(10)** | **49** | **(45-53)** | **9%** |  | **47** | **(12)** | **47** | **(39-52)** | **6%** |  | **51** | **(5)** | **52** | **(49-54)** | | **16%** |
| Hospital | 21 | (16) | 15 | (10-34) | 4% |  | 29 | (16) | 27 | (15-36) | 4% |  | 11 | (9) | 9 | (5-13) | | 4% |
| PHC | 27 | (19) | 31 | (0-44) | 5% |  | 18 | (18) | 21 | (0-34) | 2% |  | 40 | (13) | 43 | (37-49) | | 12% |
| **Hospitalization** | **309** | **(236)** | **262** | **(161-376)** | **54%** |  | **415** | **(251)** | **357** | **(273-464)** | **54%** |  | **165** | **(99)** | **154** | **(95-214)** | | **54%** |
| **Tests** | **142** | **(367)** | **69** | **(28-122)** | **25%** |  | **220** | **(469)** | **107** | **(76-165)** | **29%** |  | **37** | **(32)** | **34** | **(13-57)** | | **12%** |
| **Other treatment** | **47** | **(40)** | **58** | **(4-73)** | **8%** |  | **57** | **(41)** | **64** | **(8-87)** | **8%** |  | **34** | **(34)** | **21** | **(3-62)** | | **11%** |
| **Total Costs** | **571** | **(534)** | **486** | **(310-637)** |  |  | **764** | **(631)** | **607** | **(519-793)** |  |  | **309** | **(128)** | **300** | **(215-373)** | |  |
| **Day of hospitalization** | **33** | **(13)** | **32** | **(22-40)** |  |  | **29** | **(10)** | **27** | **(19-38)** |  |  | **38** | **(15)** | **36** | **(28-44)** | |  |
| *Note: VL treatment at the PHC consisted of the price of Glucantime and the personnel cost of the injection; capital and recurrent costs could not be estimated. VL treatment at the hospital included only the price of Glucantime as personnel, capital and recurrent costs were included in the category “hospitalization”.* | | | | | | | | | | | | | | | | | | |
